# Clinical and Genetic Spectrum of *VPS13A* Disease Across India: A Retrospective Multicentre Genetically Confirmed Cohort

**DOI:** 10.5334/tohm.1276

**Published:** 2026-10-12

**Authors:** Archita Makharia, Atif I. Shaikh, Jacky Ganguly, Ayush Agarwal, Vijayashankar Paramanandam, Mitesh Chandarana, Soaham Desai, Vaibhav Mathur, Kartika Gulati, Sanjay Pandey, Hrishikesh Kumar, Achal Kumar Srivastava, Divyani Garg

**Affiliations:** 1 Department of Neurology, All India Institute of Medical Sciences, New Delhi, India; 2 Department of Neurosciences, Christian Medical College, Vellore, India; 3 Department of Neurology, Institute of Neurosciences, Kolkata, India; 4 Department of Neurology, Apollo Hospitals, Greams Road, Chennai, India; 5 Marengo CIMS Hospital, Ahmedabad, India; 6 Zydus Hospitals, Anand, India; 7 Narayana Health, Jaipur, India; 8 Amrita Institute of Medical Sciences, Faridabad, India

**Keywords:** *VPS13A* disease, chorea-acanthocytosis, genetic spectrum, diagnostic delay, India

## Abstract

**Objectives:**

To characterize the geographic and molecular spectrum of genetically confirmed *VPS13A* disease across India and examine patterns of delay to tertiary evaluation.

**Methods:**

We conducted a multicentre retrospective study across eight tertiary movement disorder centres in India. Clinical, investigational, genetic, treatment, and follow-up data were collected.

**Results:**

Eighteen unrelated patients from eight tertiary centres were included. Chorea was the most common initial manifestation (10/18), and 8/18 patients were evaluated more than five years after symptom onset. Longer intervals to tertiary evaluation were descriptively more frequent among patients with non-choreic presentations, particularly seizures. Twenty-three distinct *VPS13A* variants were identified, predominantly loss-of-function variants, including apparently novel variants and two variants recurring in unrelated patients.

**Conclusion:**

*VPS13A* disease in this multicentre Indian cohort demonstrated substantial molecular heterogeneity, including apparently novel and recurrent variants. Longer intervals to tertiary evaluation were descriptively more frequent among patients with non-choreic presentations, particularly those with seizures, highlighting that clinicians need to maintain a high degree of suspicion for VPS13A even in such presentations.

## Introduction

Neuroacanthocytosis syndromes are rare inherited neurodegenerative disorders with movement disorders, acanthocytosis, and multisystem involvement [1]. Contemporary classification favors molecular terminology because acanthocytosis may be absent or technique-dependent [2,3]. *VPS13A* disease, traditionally termed chorea-acanthocytosis, is an autosomal recessive disorder with a broad phenotype including chorea, dystonia, tics, orofacial and lingual involvement, feeding dystonia, dysarthria, dysphagia, psychiatric and cognitive symptoms, seizures, neuropathy and myopathy [3]. Early disease may mimic McLeod neuroacanthocytosis syndrome, Huntington disease, Tourette syndrome, Wilson disease, neurodegeneration with brain iron accumulation (NBIA), epilepsy, psychiatric illness, functional tic-like disorder, or atypical parkinsonism, especially when chorea is not dominant [2].

*VPS13A* disease has been reported worldwide and is not considered restricted to a particular ethnic or geographic population, although reliable population-specific prevalence estimates are unavailable. Recurrent variants and founder effects have nevertheless been identified in selected populations. In a Japanese series of 39 molecularly confirmed patients, an exon 60–61 deletion and c.4411C>T (p.Arg1471Ter) together represented more than half of the identified mutations and were concentrated in western Japan [4]. A 37-kb deletion involving exons 70–73 has been established as a founder variant in French Canadian families, while c.2343delA was identified in nine patients from three apparently unrelated Jewish families originating from Tunisia [5,6].

Genetically confirmed reports from India remain limited and largely comprise individual families or small regional series, including four patients from three families of different Indian ethnic backgrounds [7,8,9,10]. We therefore conducted a multicentre study to characterize the geographic and molecular spectrum of *VPS13A* disease across India and to examine patterns of delay to tertiary evaluation according to the initial presenting symptom.

## Materials and Methods

We conducted a multicentre retrospective observational cohort of genetically confirmed *VPS13A*-related chorea-acanthocytosis across eight tertiary movement disorder centres in India (Supplementary Figure 1). Eligible patients were identified from clinical databases and investigator records, and data were collected between January and May 2026. All genetically confirmed cases available at participating centres were included. None of the patients included in this cohort had been previously reported in a published case report or case series. No sample-size calculation was performed. Ethical approval was obtained as per local norms (IEC #273/16.05.2023, RP-10/23, OP-20/12.01.2024). Written informed consent was obtained for publication of videos. This study is reported in accordance with the Strengthening the Reporting of Observational Studies in Epidemiology (STROBE) guideline for observational studies (Supplementary Table 1) [11].

Data were collected using a standardized proforma. Laboratory, electrophysiological, and imaging data were extracted from records and were not centrally standardized. Rubber-person gait was defined clinically as an unusual gait characterized by excessive truncal movements, intermittent lurching, and sudden buckling of the knees or trunk [12,13,14]. Imaging abnormalities were extracted from contemporaneous formal radiology reports and, where scans were available. Caudate atrophy was defined visually as reduction in the volume of the caudate head and/or body, with loss of normal caudate convexity and associated enlargement of the frontal horns of the lateral ventricles on axial or coronal images. There was no central blinded review or quantitative volumetric analysis. Acanthocyte results were abstracted from routine clinical laboratory reports performed by light microscopy. Electron microscopy was not performed, and preparation techniques and positivity thresholds were not standardized across centres. Genetic testing method, zygosity, variant location, and reported American College of Medical Genetics and Genomics (ACMG) classification were recorded. Variant localization was categorized as exonic/coding, intronic/splice-site or splice-region, mixed, or unclassifiable.

Continuous and categorical variables were summarized descriptively. Missing, unknown, or not-done values were excluded from variable-specific denominators. Diagnostic-delay comparisons were descriptive and exploratory. Selection and information bias were addressed by including all eligible genetically confirmed cases, using a standardized proforma, and reporting variable-specific denominators.

## Results

### Cohort characteristics

Eighteen genetically proven patients with *VPS13A* disease were included. Sex as recorded was male in 12 patients (66.7%) and female in 6 patients (33.3%). Mean age at presentation was 36.9 ± 7.2 years [median 35.0; range 28.0–56.0], and mean age at symptom onset was 30.0 ± 7.4 years [median 28.5; range 18.0–49.0].

Parental consanguinity was documented in 4/18 (22.2%) patients. A family history of genetically unconfirmed neurological or psychiatric symptoms in a first- or second-degree relative was present in 4/18 (22.2%) patients. None of the patients included in the present cohort were related to one another.

### Clinical Characteristics

All patients had generalized chorea, frequently accompanied by orofacial and lingual movement abnormalities. Seizures, peripheral neuropathy, psychiatric manifestations, and other movement disorders occurred in variable proportions (Table 1). The movement phenomenology is illustrated in Video 1. Formal cognitive testing was available only in a subset and showed variable impairment (Supplementary Table 2).

**Table 1:** Phenotypic spectrum of genetically confirmed *VPS13A* disease.


DOMAIN	FEATURE/PHENOMENOLOGY	TOPOGRAPHY/SUBTYPE	n (%)

Movement disorders	Chorea	Generalized	18/18 (100.0%)

		Upper face	8/18 (44.4%)

		Lower face	12/18 (66.7%)

		Lingual	11/18 (61.1%)

		Upper limb	17/18 (94.4%)

		Lower limb	17/18 (94.4%)

		Trunk/neck	16/18 (88.9%)

	Dystonia	Blepharospasm	5/18 (27.8%)

		Jaw	9/18 (50.0%)

		Lower facial	11/18 (61.1%)

		Lingual	16/18 (88.9%)

		Laryngeal	1/18 (5.6%)

		Cervical	4/18 (22.2%)

		Truncal	2/18 (11.1%)

		Lower limb	3/18 (16.7%)

		Upper limb	2/18 (11.1%)

	Tics		9/18 (50.0%)

	Ataxia		3/18 (16.7%)

	Stereotypies		3/18 (16.7%)

	Other movement disorder	Parkinsonism	2/18 (11.1%)

		Myoclonus	2/18 (11.1%)

		Akathisia	2/18 (11.1%)

		Tremor	1/18 (5.6%)

Eye movement abnormalities	Delayed vertical saccade initiation		6/18 (33.3%)

	Delayed horizontal saccade initiation		6/18 (33.3%)

	Slow/absent vertical saccades		4/18 (22.2%)

	Slow/absent horizontal saccades		4/18 (22.2%)

	Restricted vertical gaze		0/18 (0.0%)

	Restricted horizontal gaze		1/18 (5.6%)

	Ophthalmoparesis		1/18 (5.6%)

	Nystagmus		0/18 (0.0%)

Orofacial/Bulbar	Feeding dystonia		17/18 (94.4%)

	Dysarthria		16/18 (88.9%)

	Lingual dystonia		16/18 (88.9%)

	Oral Injuries		12/18 (66.7%)

	Dysphagia		4/18 (22.2%)

Psychiatric	Anxiety		9/15 (60.0%)

	Depression		8/15 (53.3%)

	Psychosis		2/16 (12.5%)

Additional neurological features	Rubber-man/woman gait		10/18 (55.6%)

	Peripheral neuropathy		12/18 (66.7%)

	Seizures		7/18 (38.9%)

	Myopathy		3/18 (16.7%)

	Pyramidal signs		2/18 (11.1%)

	Sleep abnormalities		2/18 (11.1%)

	Dysautonomia		1/18 (5.6%)

Neuroimaging findings	Caudate atrophy		14/15 (93.3%)

	Caudate signal change		6/12 (50.0%)

	Putamen signal change		5/12 (41.7%)

	Putamen atrophy		6/13 (46.2%)

	Cerebellar atrophy		4/14 (28.6%)

	Cortical atrophy		4/14 (28.6%)

	Striatal iron deposition		1/11 (9.1%)


*Denominators vary across neuroimaging findings because magnetic resonance imaging availability, sequence coverage, and interpretability differed by patient and by imaging feature; percentages were calculated using the number of patients with assessable imaging for each finding.

**Video 1:** Representative movement phenomenology in genetically confirmed *VPS13A* disease. **Case 2:** Broken horizontal and vertical smooth pursuits without ophthalmoparesis. Generalized chorea involving the bilateral upper and lower limbs, trunk, upper and lower face, with a jack-in-the-box tongue. During walking, choreiform movements were more prominent in the left upper limb, with additional lower-limb involvement. Parkinsonian gait with hypomimia was also evident. **Case 8:** Lower facial chorea with jack-in-the-box tongue, characterized by inability to sustain tongue protrusion, along with lingual dystonia. **Case 9:** Blepharospasm with a geste antagoniste, relieved by touching/bringing the hand toward the forehead. Generalized chorea involved the upper and lower face, bilateral upper and lower limbs, and trunk. Mild feeding dystonia was seen while bringing food into the mouth, without difficulty in chewing or swallowing. Hypomimia and a parkinsonian gait were present. **Case 11:** Severe oromandibular–bulbar involvement with lingual dystonia, jack-in-the-box tongue, oral injuries, drooling, marked feeding dystonia, significant dysarthria, and orofacial dystonia. Chorea involved the upper limbs, more prominent on the right, with truncal involvement and gait instability showing a rubber-woman pattern. Hypomimia/parkinsonian facial expression was also evident.

### Diagnostic Delay

Chorea was the most common initial manifestation (10/18), but non-choreic initial manifestations were also frequent and included oromandibular-bulbar, psychiatric/cognitive, epileptic, neuropathic, gait-related, and tic/dystonic presentations (Supplementary Figure 2). Median symptom duration before tertiary evaluation was 5.0 years [IQR 3.0–12.0; range 2.0–19.0], with delays extending up to 19.0 years. Eight patients (44.4%) were evaluated more than five years after symptom onset. Delayed tertiary evaluation (>5 years after symptom onset) was descriptively less frequent among patients presenting with chorea (2/10, 20.0%) than among those with non-choreic presentations (6/8, 75.0%). Six of seven patients (85.7%) with seizures were evaluated more than five years after symptom onset. Delayed evaluation also appeared more frequent with younger onset and clinical neuropathy. (Supplementary Figure 3).

### Investigations

Magnetic resonance Imaging (MRI) showed caudate atrophy in 14/15 (93.3%), with putaminal, cortical, and cerebellar atrophy/signal abnormalities in smaller subsets (Table 1). Acanthocyte testing was performed in all 18 patients, and detected in 14/18 patients (77.8%), indicating that 4 genetically confirmed patients had no documented acanthocytosis. Creatine phosphokinase (CK) was elevated in 12/13 patients (92.3%). Electrophysiology supported frequent peripheral nervous system involvement, with sensory neuropathy in 7/12 (58.3%), motor involvement in 4/12 (33.3%), axonal in 8/12 (66.7%), and demyelinating in none.

### *VPS13A* variants spectrum

Detailed variant information was available for 17 of 18 patients. Genetic testing was performed by whole-exome sequencing in 12/18 (66.7%), targeted gene panel in 3/18 (16.7%), clinical exome sequencing in 2/18 (11.1%), and single-gene study in 1/18 (5.6%) patients; variants were homozygous in 9/18 (50.0%) and compound heterozygous in 8/18 (44.4%) patients, with zygosity not available for 1/18 (5.6%) (Supplementary Figure 4). The molecular spectrum was heterogeneous, with variants distributed across *VPS13A* and comprising frameshift, nonsense, canonical splice-site or splice-region, in-frame deletion, missense, and multiexon deletion variants (Figure 1 and Table 2). Several variants were absent from the population and clinical databases reviewed and appeared to be previously unreported. Two variants were identified independently in more than one unrelated patient: the canonical splice-site variant c.144+1G>A in Patients 3 and 15 and the frameshift variant c.3904delG in Patients 7 and 8. The remaining identified variants were each observed in a single patient.

**Figure 1:**
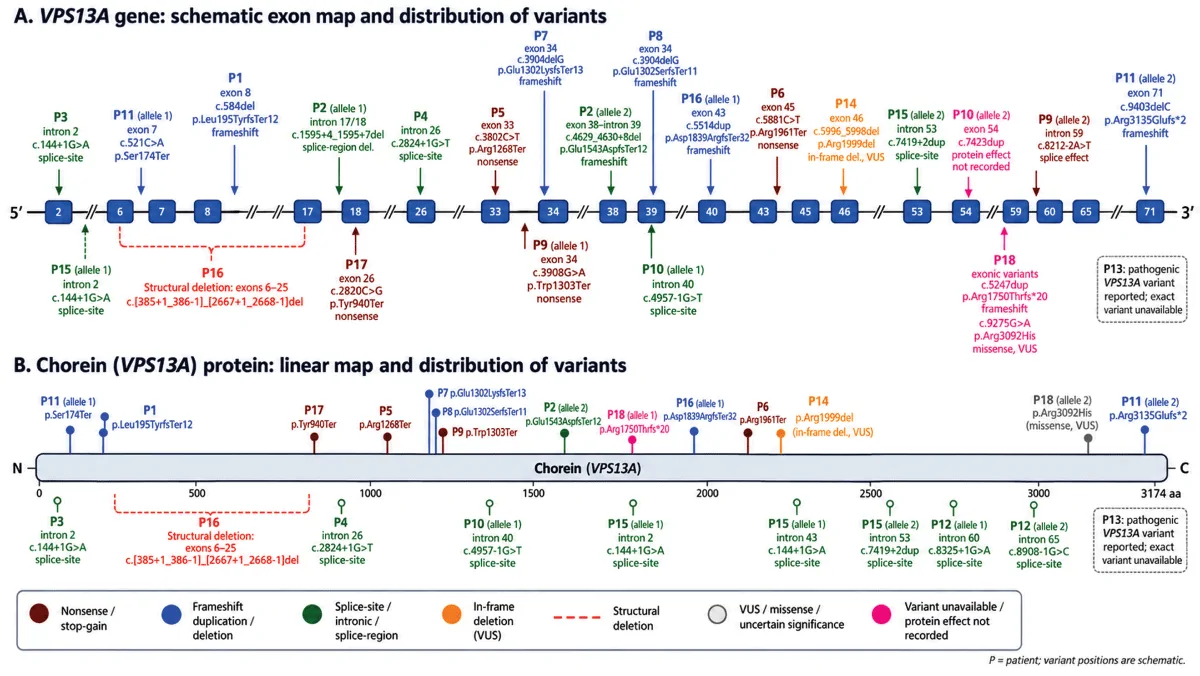
*VPS13A* gene and chorein protein variant map. Schematic localization of recorded *VPS13A* variants across the gene and chorein protein. Positions are approximate. P, patient; VUS, variant of uncertain significance.

**Table 2:** *VPS13A* variant spectrum, testing method, zygosity, localization, and ACMG classification in genetically confirmed *VPS13A* disease.


PATIENT ID	GENETIC TEST	ZYGOSITY	REFSEQ	*VPS13A* VARIANT(S)	PROTEIN CHANGE	VARIANT CLASS/LOCALIZATION	ACMG/SOURCE CLASSIFICATION	GNOMAD

Patient 1	Whole-exome sequencing	Homozygous	NM_033305.3	Exon 8 c.584del	p.Leu195TyrfsTer12	Exonic/coding; frameshift	Likely pathogenic (PVS1, PM2)	Novel

Patient 2	Whole-exome sequencing	Compound heterozygous	NM_033305.3	c.1595 + 4_1595 + 7del/ c.4629_4630 + 8del	Not available/ p.Glu1543AspfsTer12	Exon-Intron junction 17 deletion/ Exon 38-intron 39 splice junction deletion	VUS (Splice AI 0.99)/ Likely pathogenic (Splice AI score 1.00)	Novel

Patient 3	Targeted gene panel	Homozygous	NM_033305.3	Intron 2 c.144+1G>A	Not available	Intronic/splice-site	Likely pathogenic (Splice AI score 0.99)	Novel

Patient 4	Whole-exome sequencing	Homozygous	NM_033305.3	Intron 26 c.2824+1G>T	Not available	Intronic/splice-site	Likely pathogenic (Splice AI score: 1.00)	Novel

Patient 5	Whole-exome sequencing	Homozygous	NM_033305.3	Exon 33 c.3802C>T	p.Arg1268Ter	Exonic/coding; nonsense	Pathogenic (PVS1, PM2)	Minor allele frequency 0.004% (gnomAD) Not reported in SAS gnomAD Reported in ClinVar 2184590

Patient 6	Whole-exome sequencing	Homozygous	NM_033305.3	Exon 45 c.5881C>T	p.Arg1961Ter	Exonic/coding; nonsense	Pathogenic (PVS1, PM2, PM3)	Minor allele frequency 0.006% (gnomAD) Minor allele frequency (MAF): 1.1 × 10^–5^ in SAS gnomAD Reported in ClinVar 1458994

Patient 7	Whole-exome sequencing	Homozygous	NM_033305.3	Exon 34 c.3904delG	p.Glu1302 LysfsTer13	Exonic/coding; frameshift	Pathogenic (PVS1, PM2, PP4)	Minor allele frequency 1.24e-6 (gnomAD) Not reported in SAS gnomAD Absent in ClinVar

Patient 8	Targeted gene panel	Homozygous	NM_033305.3	Exon 34 c.3904delG	p.Glu1302SerfsTer11	Exonic/coding; frameshift	Pathogenic (PVS1, PM2, PP4)	Minor allele frequency 1.24e-6 (gnomAD) Not reported in SAS gnomAD Absent in ClinVar

Patient 9	Whole-exome sequencing	Compound heterozygous	NM_033305.3	Exon 34 c.3908G>A/Intron 59 c.8212-2A>T	p.Trp1303Ter/splice effect	Mixed exonic + intronic/splice-region	Pathogenic (PVS1, PM2,PM3)/Pathogenic (PVS1, PM2, PM3)	Novel ClinVar 2064491/Novel

Patient 10	Whole-exome sequencing	Compound heterozygous	NM_033305.3	Intron 40 c.4957-1G>T/exon 54 c.7423dup	Splice effect/not recorded	Mixed exonic + intronic/splice-region	Likely pathogenic (PVS1, PM2) (Splice AI score 0.98)	Novel

Patient 11	Whole-exome sequencing	Compound heterozygous	NM_033305.3	Exon 7 c.521C>A/exon 71 c.9403delC	p.Ser174Ter/p.Arg3135Glufs*2	Exonic/coding; nonsense + frameshift	Likely pathogenic (PVS1, PM2)	Novel

Patient 12	Clinical exome sequencing	Compound heterozygous	NM_033305.3	Intron 60 c.8325+1G>A/intron 65 c.8908-1G>C	Not available	Intronic/splice-site	Likely pathogenic (PVS1, PM2, PP5) (Splice AI score 1.00)/Likely pathogenic (PVS1, PM2) (Splice AI score 0.84)	Minor allele frequency 5.77e-6 in gnomAD ClinVar 1347984 (classified variably as likely pathogenic and VUS)/Novel

Patient 13	Targeted gene panel	Not available	NM_033305.3	Not available	Not available	Not available	Pathogenic	–

Patient 14	Clinical exome sequencing	Homozygous	NM_033305.3	Exon 46 c.5996_5998del	p.Arg1999del	Exonic/coding; in-frame deletion	Pathogenic (PVS1, PM2, PP4)	Novel

Patient 15	Whole-exome sequencing	Compound heterozygous	NM_033305.3	Intron 2 c.144+1G>A/Intron 53 c.7419 + 2dup	Not available	Intronic/splice-site	Likely pathogenic (Splice AI score 0.99)/ Pathogenic (Splice AI score 0.88)	Novel

Patient 16	Whole-exome sequencing	Compound heterozygous	NM_033305.3	Exon 43 c.5514dup/exons 6–25 deletion c.[385 + 1_386-1]_[2667 + 1_2668-1]del	p.Asp1839ArgfsTer32/structural deletion	Exonic/coding + structural deletion	Pathogenic (PVS1, PM2, PM3)/Pathogenic (PVS1, PM2, PM3)	Novel

Patient 17	Whole-exome sequencing	Homozygous	NM_033305.3	Exon 26 c.2820C>G	p.Tyr940Ter	Exonic/coding; nonsense	Pathogenic (PVS1, PM2, PP4)	Novel

Patient 18	Single-gene study	Compound heterozygous	NM_033305.3	Exonic c.5247dup/c.9275G>A	p.Arg1750Thrfs*20/ p.Arg3092His	Exonic/coding	Pathogenic (PVS1, PM2, PM3)/VUS (PM2, PM3, PP3)	Novel/Minor allele frequency (0.0004%) in gnomAD Absent in SAS gnomAD Reported in ClinVar 3603267


*ACMG = American College of Medical Genetics and Genomics; ClinVar = clinical variant archive; gnomAD = Genome Aggregation Database; RefSeq = Reference Sequence; SAS = South Asian; VUS = variant of uncertain significance. ACMG evidence codes are shown as reported in clinical genetic reports.

### Treatment and Follow-Up

Treatments were individualized and are summarized in Supplementary Table 3. At last follow-up, 12/17 patients remained independent in activities of daily living. Follow-up functional data demonstrated a difference between feeding and speech outcomes: 16/17 (94.1%) patients retained feeding ability, whereas only 6/17 (35.3%) had comprehensible speech. All patients continued oral feeding with dietary texture modification and/or compensatory swallowing manoeuvres .None received nasogastric or gastrostomy feeding. Enteral feeding was recommended by the treating clinician for 10/18 patients to support nutritional adequacy, but none accepted it. Reported reasons, which were not mutually exclusive, included perceived stigma (8/10), fear of tube placement or feeding (6/10), and inadequate local facilities for tube-feeding care (2/10). Age at onset and symptom duration were similar between groups, but dependent patients descriptively showed a greater burden of seizures, peripheral neuropathy, dysphagia, and incomprehensible speech (Table 3).

**Table 3:** Comparison of patients independent versus dependent in activities of daily living at last follow-up.


FEATURE	INDEPENDENT (n = 12)	DEPENDENT (n = 5)

Median age at onset, years	28.5 (18–49)	28.0 (25–44)

Median symptom duration, years	5 (2–20)	6 (2–12)

Choreic onset (first symptom)	5/12 (41.7%)	4/5 (80.0%)

Seizures	4/12 (33.3%)	3/5 (60.0%)

Peripheral neuropathy	7/12 (58.3%)	4/5 (80.0%)

Dysphagia	2/12 (16.7%)	2/5 (40.0%)

Oral injuries	7/12 (58.3%)	4/5 (80.0%)

Rubber-man/woman gait	8/12 (66.7%)	3/5 (60.0%)

Comprehensible speech	5/12 (41.7%)	1/5 (20.0%)


Values are median (range) or n/N (%). Functional data were available for 17/18 patients (one patient had no follow-up). Continuous values were similar between groups; percentages were calculated within each group. Given the small dependent subgroup (n = 5), comparisons are descriptive and no statistical testing was performed.

## Discussion

India remains an underexplored setting for *VPS13A* disease, with published evidence largely confined to individual reports and small familial or regional series [7,8,9,10]. This multicentre cohort broadens its documented geographic distribution across India and adds substantially to the limited molecular data available from South Asian populations. However, the observed distribution represents tertiary-referral ascertainment rather than regional prevalence. Geographic clustering or under-representation may reflect differences in clinical recognition, access to molecular testing, referral pathways, and publication practices. Because ancestry and haplotypes were not systematically examined, the present study cannot establish founder effects or population-specific enrichment within India.

The principal strength of this cohort lies in its molecular data. Twenty-three distinct *VPS13A* variants were identified across 17 genotyped patients, and some appeared to be previously unreported, substantially extending the known allelic spectrum of *VPS13A* disease beyond the handful of variants previously described from India. This scale of novel-variant discovery, together with two variants recurring independently in unrelated patients empahsizes the value of systematic multicentre molecular characterisation in a population that remains markedly underrepresented in existing *VPS13A* variant databases. The relatively high representation of splice-altering variants and the identification of a multiexon deletion support comprehensive molecular testing that includes splice-junction coverage and copy-number analysis [15].For non-canonical splice-region variants and variants of uncertain significance, segregation and RNA studies may demonstrate aberrant splicing, while erythrocyte chorein Western blotting can provide complementary functional evidence of *VPS13A* dysfunction [16,17,18]. However, normal chorein expression does not exclude *VPS13A* disease, because some pathogenic missense variants may impair protein function without causing protein misfolding or degradation, while small deletions may produce a truncated protein with electrophoretic mobility similar to that of the wild-type protein [13].

Alongside this molecular contribution, the cohort provides descriptive evidence that diagnostic delay differs systematically by presenting phenotype, a clinically actionable finding. Chorea may prompt earlier consideration of a hereditary neurodegenerative disorder, whereas patients presenting with seizures may initially remain within epilepsy-care pathways until additional movement or multisystem manifestations emerge [6,19]. Psychiatric, neuropathic, or other non-choreic presentations may similarly lead patients through separate specialist pathways before the unifying disorder is recognized. These comparisons are descriptive and based on small numbers, but they identify a clinically relevant hypothesis for evaluation in larger cohorts.

The measured interval should not be interpreted as equivalent to the time to definitive molecular diagnosis or as an intrinsic property of *VPS13A* disease. It is influenced by geographical and financial access, referral practices, availability of genetic testing, and healthcare-seeking behaviour [20,21]. Predominantly out-of-pocket genetic testing may further restrict ascertainment in India, although socioeconomic status and the source of funding for testing were not systematically recorded. The findings nevertheless suggest that awareness of *VPS13A* disease among epileptologists, psychiatrists, and neuromuscular specialists, not only movement-disorder specialists, may facilitate earlier referral of patients with evolving non-choreic presentations.

Despite clinically significant bulbar dysfunction, all patients continued oral feeding, including those for whom enteral feeding had been recommended. Non-acceptance appeared to reflect overlapping psychosocial and healthcare-access barriers, particularly perceived stigma, fear, and limited local support for tube-feeding care. However, nutritional adequacy, aspiration risk, formal swallowing assessments, reasons for recommending enteral feeding, and patient goals of care were not systematically recorded. These findings should therefore not be interpreted as demonstrating preserved swallowing safety or adequate nutrition among patients who continued oral intake.

Caudate-predominant atrophy was the most frequent imaging finding, whereas putaminal abnormalities and cortical and cerebellar atrophy occurred in smaller subsets. These findings are supportive but not specific and may overlap with Huntington disease and other hereditary choreas [22,23]. Functional imaging studies have also demonstrated striatal metabolic, perfusion, and dopaminergic abnormalities in *VPS13A* disease, but these have not been validated as reliable discriminators from Huntington disease [24,25,26]. Neuroimaging should therefore be interpreted in the clinical context, with definitive distinction dependent on molecular testing.

Acanthocytes were not documented in four genetically confirmed patients despite testing. This finding should be interpreted cautiously because smear preparation, microscopy methods, positivity thresholds, and sample-handling procedures were not standardized across participating centres. Acanthocytosis is a supportive, technique-dependent finding, and the proportion of acanthocytes has not shown a consistent relationship with disease severity [27,28]. Accordingly, the absence of documented acanthocytosis should not preclude molecular testing when *VPS13A* disease is clinically suspected. Together, these clinical, imaging, and molecular observations support a staged diagnostic approach in resource-variable settings, as summarized in Supplementary Figure 5.

This study is limited by its retrospective design, modest sample size, and non-standardized clinical, laboratory, and imaging assessments. Neuroimaging was interpreted qualitatively at individual centres without centralized review or predefined quantitative thresholds. Acanthocyte-testing methods could not be compared across centres, and validated longitudinal measures of movement, speech, swallowing, functional progression, and treatment response were unavailable. Recruitment from tertiary movement-disorder centres and the requirement for molecular confirmation introduce referral and ascertainment biases and may exclude patients without access to specialist care or genetic testing. Furthermore, socioeconomic, ancestry, and haplotype data were not systematically collected. Formal swallowing assessments, aspiration events, nutritional adequacy, concurrent oral and enteral feeding plans, and goals-of-care considerations were not systematically collected. Despite these limitations, this multicentre cohort broadens the geographic and molecular spectrum of *VPS13A* disease reported from India and identifies apparently novel and recurrent variants, while its descriptive delay patterns highlight non-choreic presentations, particularly seizures, as potential points for improving earlier recognition.

## Additional File

The additional file for this article can be found as follows:

10.5334/tohm.1276.s1 Supplementary File. Supplementary Figures 1–5 and Supplementary Tables 1–3.
