# Supplementary material for: Clinical and Genetic Spectrum of VPS13A Disease Across India: A Retrospective Multicentre Genetically Confirmed Cohort
Source: Tremor Other Hyperkinet Mov (N Y). 2026 Oct 12;16:56. doi: 10.5334/tohm.1276 (PMC13674526; doi:10.5334/tohm.1276)
Supplement: Supplementary file 1 [file tohm-16-1-1276-s1.pdf]

Supplementary File 1

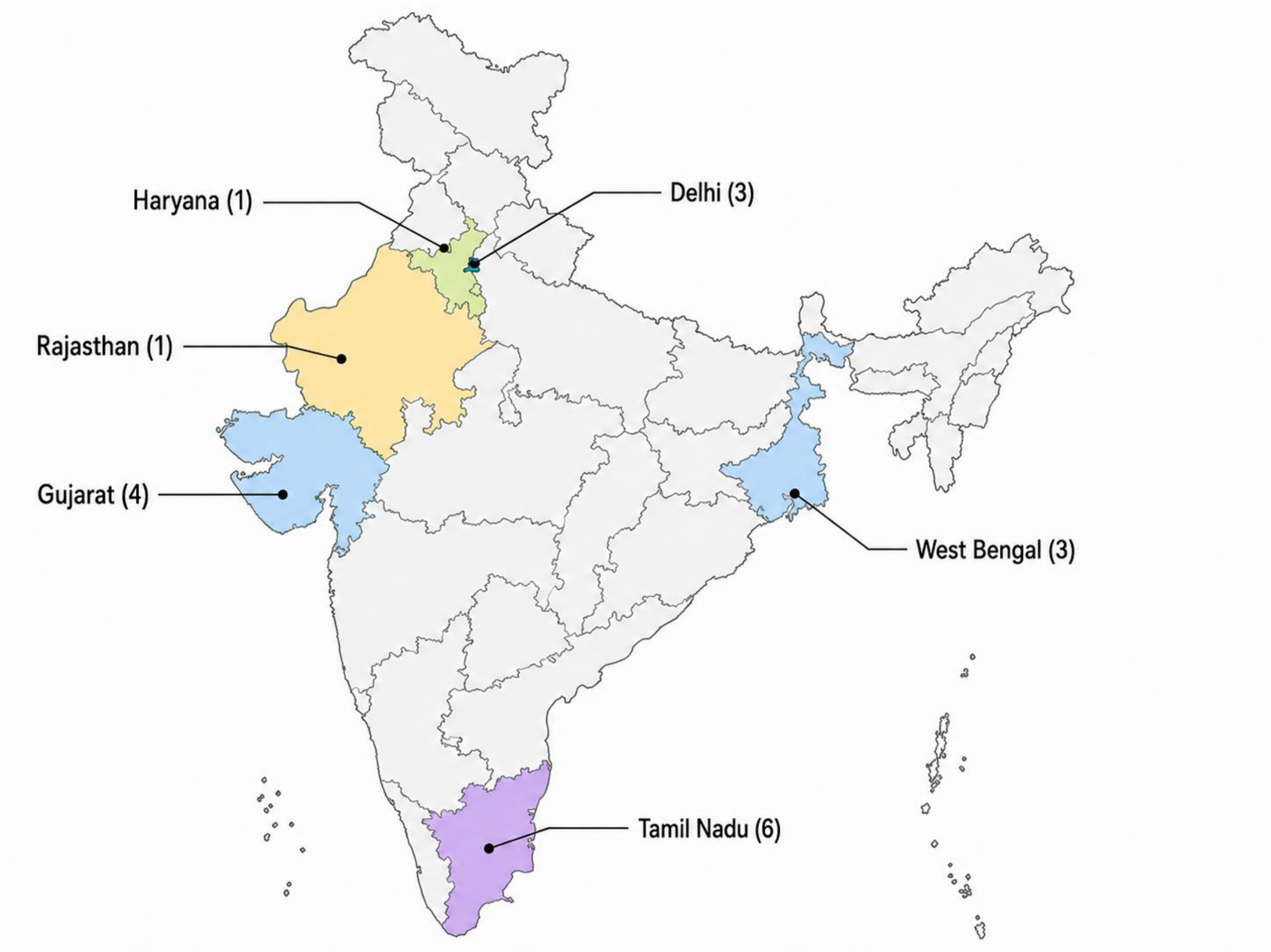

*Numbers in parentheses indicate the number of patients contributed by each state/region.

Supplementary Figure 1. Geographical distribution of participating centres

Supplementary File 1

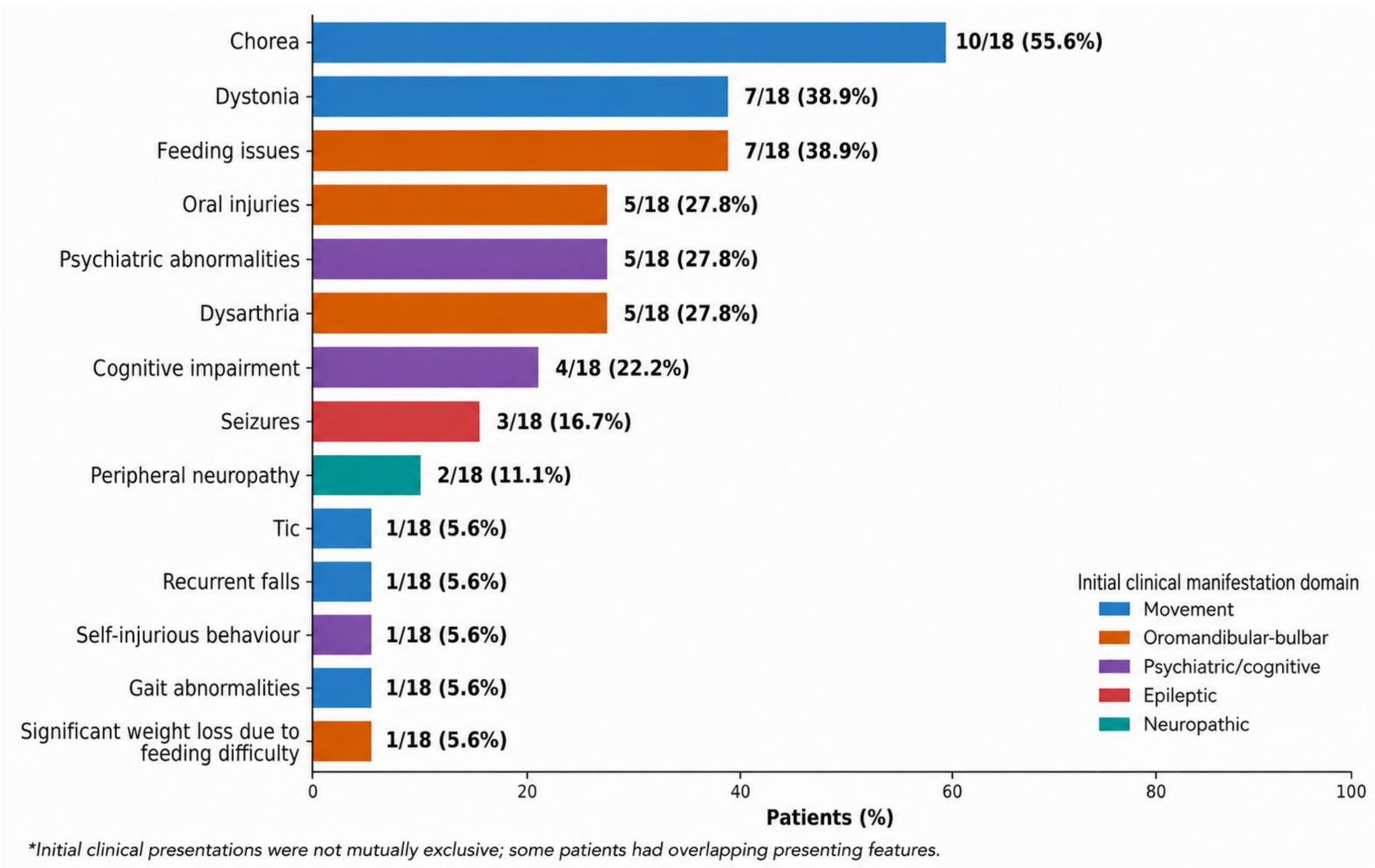

Supplementary Figure 2. Initial Clinical Manifestations

Supplementary File 1

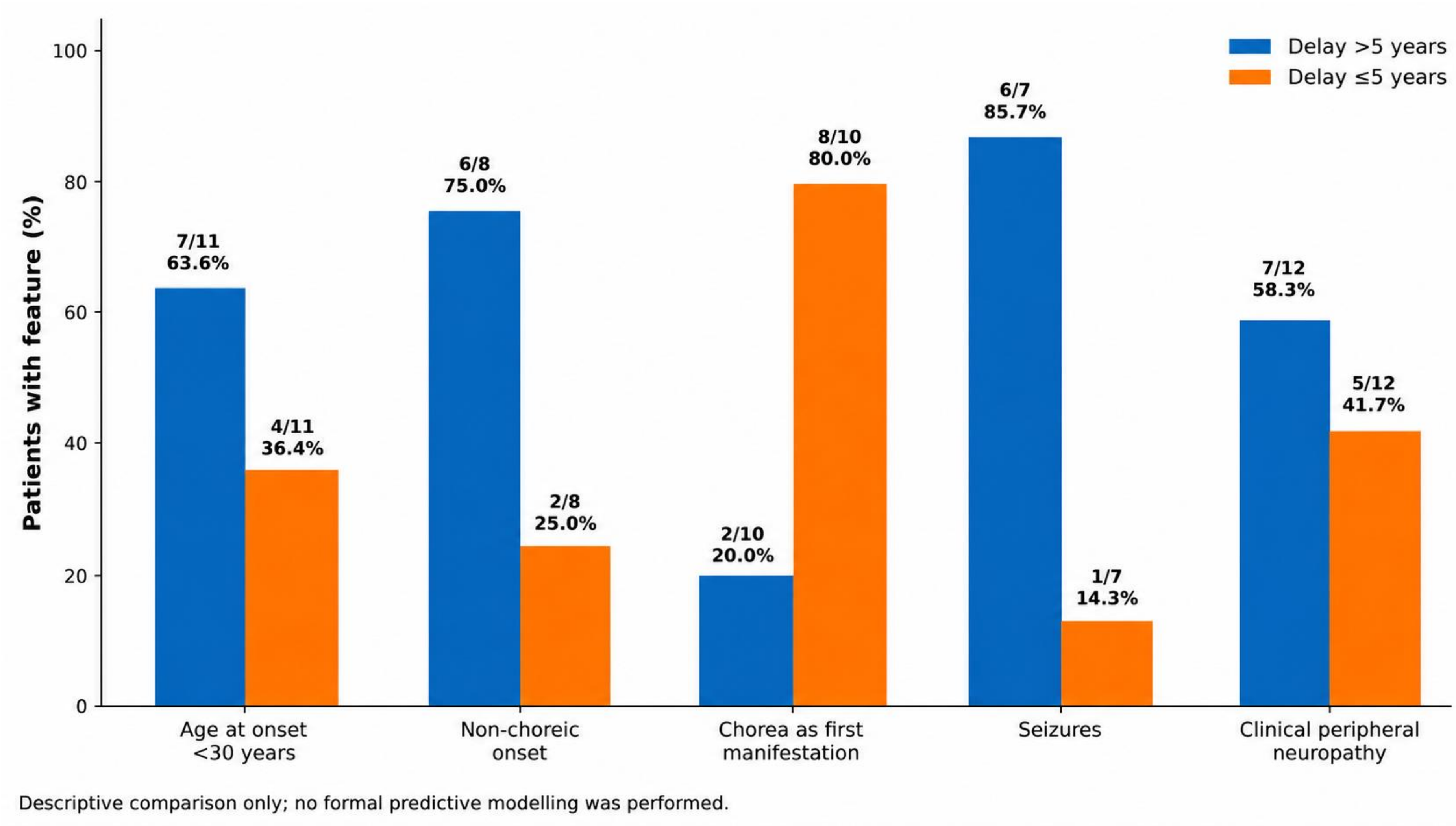

Supplementary Figure 3. Descriptive comparison of clinical features by diagnostic-delay status

Supplementary File 1

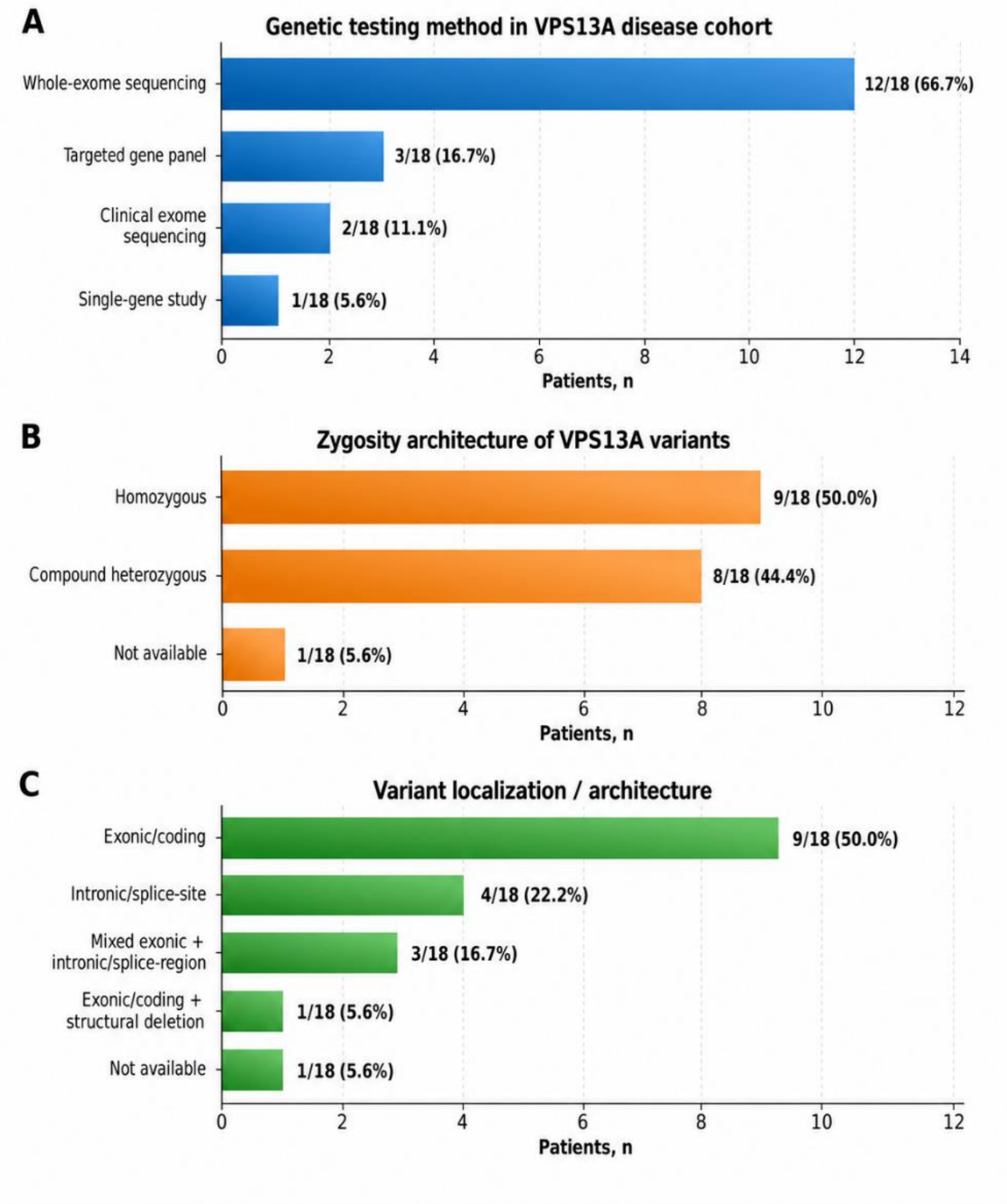

Supplementary Figure 4. Genetic Testing Methods and *VPS13A* Variant Architecture

Supplementary File 1

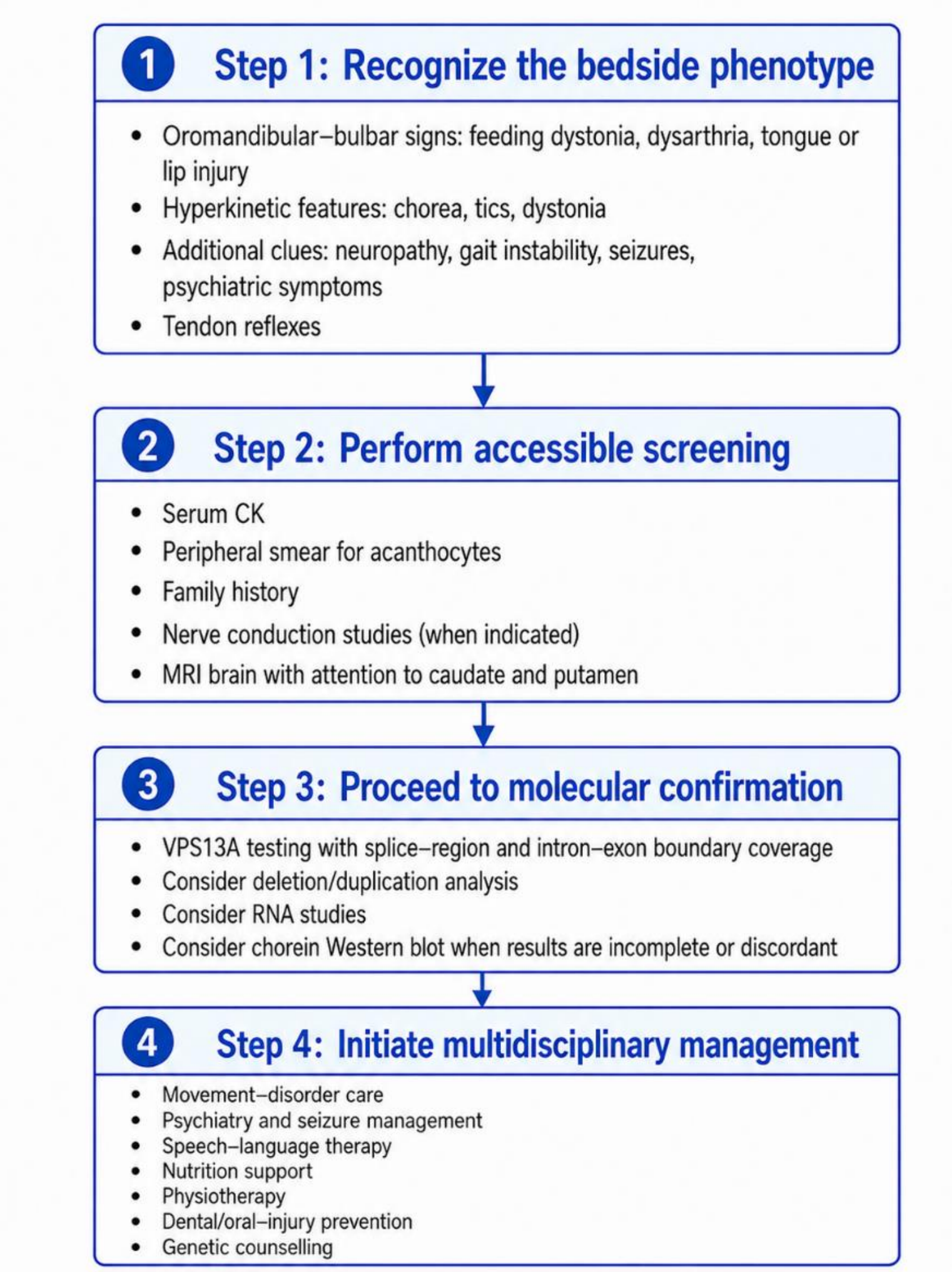

Supplementary Figure 5. Staged diagnostic approach to suspected *VPS13A* disease in resource-variable settings

Supplementary File 1

Supplementary Table 1: STROBE checklist

**Item No.**	**STROBE recommendation**	**Reported location in manuscript**
**1(a)**	Indicate the study design with a commonly used term in the title or abstract.	Title; Abstract, Methods
**1(b)**	Provide an informative and balanced summary of what was done and found.	Abstract
**2**	Explain the scientific background and rationale for the investigation.	Introduction
**3**	State specific objectives, including any prespecified hypotheses.	Introduction
**4**	Present key elements of study design early in the paper.	Methods
**5**	Describe the setting, locations, and relevant dates, including recruitment, exposure, follow-up, and data collection periods.	Methods; Supplementary File 1, Figure 1
**6(a)**	Give eligibility criteria and sources/methods of participant selection.	Methods
**6(b)**	For matched studies, give matching criteria and number of exposed/unexposed participants.	Not applicable; this was not a matched study
**7**	Clearly define outcomes, exposures, predictors, potential confounders, and effect modifiers.	Methods; Results
**8**	For each variable of interest, give sources of data and details of assessment methods.	Methods
**9**	Describe efforts to address potential sources of bias.	Methods; Discussion, limitations

Supplementary File 1

**10**	Explain how the study size was arrived at.	Methods
**11**	Explain how quantitative variables were handled in the analyses.	Methods
**12(a)**	Describe all statistical methods, including those used to control for confounding.	Methods
**12(b)**	Describe methods used to examine subgroups and interactions.	Methods; diagnostic-delay comparisons described as exploratory
**12(c)**	Explain how missing data were addressed.	Methods
**12(d)**	For cohort studies, explain how loss to follow-up was addressed.	Results; follow-up denominators reported using available data
**12(e)**	Describe any sensitivity analyses.	Not performed; descriptive rare-disease cohort
**13(a)**	Report numbers of individuals at each stage of the study.	Results
**13(b)**	Give reasons for non-participation at each stage.	Not applicable; retrospective cohort of all available genetically confirmed cases
**13(c)**	Consider use of a flow diagram.	Not used; cohort size and inclusion are described in text
**14(a)**	Give characteristics of study participants.	Results; Table 1
**14(b)**	Indicate number of participants with missing data for each variable.	Methods; Results using variable-specific denominators
**14(c)**	Summarize follow-up time.	Results
**15**	Report numbers of outcome events or summary measures over time.	Results; Supplementary File 1

Supplementary File 1

**16(a)**	Give unadjusted estimates and, if applicable, confounder-adjusted estimates with precision.	Not applicable; descriptive study without adjusted modelling
**16(b)**	Report category boundaries when continuous variables were categorized.	Methods; diagnostic delay defined as >5 years
**16(c)**	If relevant, translate estimates of relative risk into absolute risk.	Not applicable
**17**	Report other analyses, including subgroup and sensitivity analyses.	Results; diagnostic-delay comparisons described as exploratory
**18**	Summarize key results with reference to study objectives.	Discussion
**19**	Discuss limitations, including sources of potential bias or imprecision.	Discussion
**20**	Give a cautious overall interpretation considering objectives, limitations, and other evidence.	Discussion; Conclusion
**21**	Discuss generalisability of the study results.	Discussion; limitations
**22**	Give source of funding and role of funders.	Title page/declarations: Funding Source: None

Supplementary File 1

Supplementary Table 2. Cognitive screening scores in patients with available formal testing

Cognitive measure	Patients tested, n	Mean ± SD	Median	Range
**MoCA**	8	27.2 ± 3.7	29.5	21-30
**MMSE**	8	25.8 ± 5.4	28.0	15-30
**FAB**	4	10.2 ± 2.2	10.0	8-13

*MoCA = Montreal Cognitive Assessment, MMSE = Mini-Mental State Examination, FAB = Frontal Assessment Battery

Supplementary File 1

Supplementary Table 3. Symptomatic treatments used in the cohort

Treatment	n/N (%)
Tetrabenazine	16/18 (88.9%)
Trihexyphenidyl	11/18 (61.1%)
Clonazepam	12/18 (66.7%)
Botulinum toxin	7/18 (38.9%)
Antiseizure medications	5/18 (27.8%)
Antidepressant/SSRI	5/18 (27.8%)
Amantadine	1/18 (5.6%)
Dental guard	1/18 (5.6%)
Quetiapine	5/18 (27.8%)
Risperidone	4/18 (22.2%)
Haloperidol	3/18 (16.7%)
Aripiprazole	2/18 (11.1%)
Olanzapine	0/18 (0%)

Treatments were not mutually exclusive, and individual patients could receive more than one medication during follow-up.
